# Comparing Pediatric 72-Hour Emergency Department Returns: General vs Pediatric Emergency Departments

**DOI:** 10.5811/westjem.21302

**Published:** 2025-08-29

**Authors:** Dana Libov, Mark Zocchi, Arvind Venkat, Timothy Ruttan, Coburn Allen, Matthew Wilkinson, US Acute Care Solutions Research Group

**Affiliations:** * Northwell Health, New Hyde Park, New York; † Brandeis University, Heller School for Social Policy and Management, Waltham, Massachusetts; ‡ Allegheny Health Network, Department of Emergency Medicine, Pittsburgh, Pennsylvania; § University of Texas at Austin Dell Medical School, Department of Pediatrics, Austin, Texas; || US Acute Care Solutions, Canton, Ohio

## Abstract

**Introduction:**

There is limited data comparing pediatric return visits between pediatric emergency departments (PED) and general EDs. We hypothesized that the 72-hour return rate is higher for patients discharged from general EDs than from PEDs.

**Methods:**

We analyzed all PED visits in a large, national emergency medical group that had a repeat ED visit to the same site within 72 hours between 2016–2019. Associated visit- and facility-level characteristics analyzed in the model included patient age, Emergency Severity Index and triage level, sex, insurance type, categorized reason for visit, facility type, facility size, trauma status, teaching status, year, and month. Diagnostic categories were defined using the Agency for Healthcare Research and Quality clinical classification software for diagnosis codes. The outcome variable was 72-hour returns. We analyzed returns at the visit-level using descriptive statistics and at the facility-month level using logistic regression to adjust for potential confounders.

**Results:**

A total of 2,588,680 pediatric visits were included: 1,821,800 from 137 general EDs and 766,880 from 7 PEDs. The proportion of children returning to a PED within 72 hours was 1.1 percentage points higher than at a general ED (3.5% vs. 2.4%). The adjusted odds ratio for a 72-hour return visit was 1.3 (confidence interval 1.15–1.38) in PEDs compared to general EDs.

**Conclusion:**

Pediatric patients discharged from PEDs had a higher rate of 72-hour return visits than those discharged from general EDs. These findings merit further investigation into factors driving these differences to identify best practices and optimize care across ED settings.

## INTRODUCTION

More than 80% of children who seek emergency medical treatment are seen in a general emergency department (ED).^1^ Despite the large total volume of patients visiting general EDs, the individual facilities themselves often see very few pediatric patients, with some GEDs seeing as few as ≤10 patients per day.^2^ However, most pediatric patients are seen in general EDs, which on average treat <14 children per day.^3^ Given the variability in pediatric exposure, there is concern that the care provided to pediatric patients in general EDs may be suboptimal when compared to specialized pediatric EDs (PED) that are attached to dedicated children’s hospitals. The 72-hour return visit is frequently used as an indicator of quality in emergency medical care. While these return visits may represent progression of disease and be unavoidable or even desired in some instances, to the extent that these visits may be preventable and/or unnecessary they “represent an important quality indicator and benchmark for emergency department care.”^5^ In addition, potentially avoidable return visits have significant implications on use of resources, healthcare costs and reimbursements, and ED crowding.^5^

There are limited data comparing the frequency of ED return visits for pediatric patients by facility types, specifically PEDs as opposed to the general EDs that see most pediatric patients in the United States. We aimed to investigate the differences in 72-hour ED returns between general EDs and PEDs. We hypothesized that the rate of 72-hour return visits for children would be greater in general EDs than in PEDs based on the difference in frequency of pediatric visits and disparities in pediatric resources, as well as different levels of pediatric-specific expertise and training in facility staff.

## METHODS

This was a cross-sectional study of pediatric ED visits treated at EDs staffed by a national emergency medicine staffing group over a four-year period (2016–19). The private dataset is part of the billing database of the staffing company and includes 137 general EDs and 7 PEDs in 22 states. Data elements in this dataset are automatically extracted from electronic health records and billing data at each site. Coding specialists employed by the national group audited the data. The study was approved by the Institutional Review Board of Allegheny Health Network.

We extracted visit characteristics for all patients <18 years of age as this was a study of pediatric patients presenting to the ED. Most of the pediatric hospitals included in this study admit patients up until their 18th birthday; so to ensure uniformity we used this age cutoff for all hospitals. Each visit’s primary diagnosis, per the *International Classification of Diseases, Rev. 10* (ICD-10), was assigned to a clinical classification category using the Agency for Healthcare Quality Clinical Classifications Software Refined (CCSR v2019.1). The CCSR aggregates more than 70,000 ICD-10 diagnosis codes into 530 clinically meaningful categories. Members of the study team (DL and TR) then mapped each of these CCSR categories to 52 pediatric emergency reason-for-visit clusters. Additional visit characteristics extracted included age, sex, Emergency Severity Index (ESI) triage level, payor source (commercial, Medicaid, Medicare, self-pay, and other), disposition (discharged, admitted, transferred, etc), and primary treating clinician type (physician or advanced practice practitioner). Characteristics of the EDs included ED size (based on total annual adult and pediatric volume) and ED location classified using urban-rural classification codes (eg, large central metro, large fringe metro, medium metro, and small and non-metro).^4^

A 72-hour return was defined as a subsequent ED visit to the same ED within 72 hours following discharge from an original ED visit (the index visit). The discharge date must have occurred between January 4, 2016–December 28, 2019 to allow a 72-hour follow-up period for all index visits. We excluded as index visits patients assigned a triage ESI level of 1 or 2 and those admitted to an inpatient unit or transferred from the ED, as well as those who left without being seen or against medical advice, as these patient groups could be consistently tracked throughout our database. As a result, all index visits had a disposition of discharged with an ESI triage level ≥ 3.

**Population Health Research Capsule**

What do we already know about this issue?

*Most pediatric visits occur at general EDs as opposed to pediatric EDs, but little is known about how return rates differ by ED type.*

What was the research question?

*Do 72-hour return rates differ for pediatric patients seen at general vs pediatric EDs?*

What was the major finding of the study?

*Pediatric EDs had higher adjusted 72-hour return rates vs general EDs (3.5 vs. 2.4%, aOR 1.3, 95% CI 1.15–1.48, P < .001).*

How does this improve population health?

*These findings highlight opportunities to improve discharge planning, especially for pediatric patients presenting with high-risk conditions.*

We used descriptive statistics (counts, proportions) to compare index visit and facility characteristics at PEDs and general EDs. To explore differences in 72-hour return rates between general EDs and PEDs, we used logistic regression models to calculate the proportional difference in return rates by visit characteristics and facility characteristics with standard errors adjusted for clustering at the facility level. To estimate the overall difference in return rates between general EDs and PEDs while controlling for potential confounding variables, we then used a multivariable logistic regression model also with cluster-adjusted standard errors. The model included the same index visit and facility characteristics described previously as well as dummy variables for the calendar year and month of visit.

## RESULTS

A total of 2,588,680 patients were included across all EDs, with 1,821,800 from 137 general EDs and 766,880 from 7 PEDs. The most common reasons to present to an ED—fever, upper respiratory infection (URI), abdominal pain, rash—were similar across facilities. Congestion and URIs, for example, were 18.9% of visits in general EDs and 22.2% in PEDs (Table 1). The proportion of children returning to a PED within 72 hours was higher than those returning to a general ED (3.5% vs 2.3%), yielding an unadjusted odds (aOR) ratio of 1.48 (confidence interval [CI] 1.35–1.64). When adjusted for sex, age, payor mix, ESI level, pediatric emergency reason-for-visit clusters, clinician type, ED size, ED location, and year and month of visit, the aOR for a return visit was 1.30 (CI 1.15–1.48), suggesting the adjusted odds of re-presentation within 72 hours was 30% higher for children presenting to a PED than those seen at a general ED. This relative difference in 72-hour return visits was roughly consistent across ESI levels 3, 4 and 5 as well as facility size, sex, and age (Table 2).

The initial study design excluded ESI levels 1 and 2, as noted in the methods section, to focus on patients with more comparable acuity across ED types. We observed many ESI level 1 and 2 patients discharged from general EDs compared to PEDs. This discrepancy raises the concern that factors unrelated to patient characteristics, such as overtriage due to limited familiarity with pediatric patients in general EDs, may be driving this difference. Importantly, even when we performed an analysis including all ESI levels, the aOR remained consistent, suggesting that the observed trends were not solely driven by the distribution of ESI levels. The inclusion of these factors highlights the need for further investigation into their contributions to the variability in return visits.

Additional analysis revealed that the percentage of return visits was consistent across different facility sizes and patient age groups, indicating that these factors did not account for the higher rate of return visits observed at PEDs compared to general EDs. What remains unaccounted for is whether PEDs are disproportionately managing more complex cases, reflecting their role as specialized facilities.

## DISCUSSION

We sought to compare 72-hour return visit rates between PEDs and general EDs within a large, national network of EDs. Despite the differences in pediatric volume and presumably familiarity, we found that children seen in a PED were more likely to return to the same ED within 72 hours as compared to those cared for in a general ED. This finding was contrary to our initial study hypothesis. These findings held true across the most common conditions as well as younger age groups, all of which were less likely to re-present to a general ED than to a PED.

Studies dating back to at least 2004 have explored return rates in pediatric emergency medicine, often in the context of their use as a quality measure or initiatives to reduce these rates.^5^,^13^,^14^ Additionally, analyses using National Hospital Ambulatory Medical Care Survey data have consistently reported pediatric return rates similar to those observed in our study.^15^ These findings indicate that return rates for pediatric emergency visits are not only well-documented but also consistent across different healthcare settings. However, to differentiate our work, we leveraged the strength of a large, networked ED system with detailed patient- and facility-level data and explored the differences among general and PEDs. This approach allowed us to explore nuanced differences in return visit rates by facility type, patient age, and specific diagnostic categories. Our analysis revealed that PEDs exhibited higher return rates compared to general EDs, even after adjusting for potential confounders such as triage level and facility size.

We also observed that younger children were more likely to re-present to PEDs than general EDs and that the younger the child, the larger the absolute difference in return visit rates. The reason for this is unclear; our dataset does have limitations as we were unable to analyze the evaluation done on individual patients. For example, it may be that children in the general ED received more testing such as labs and imaging at the initial ED visit, which would have provided general emergency clinicians with more data to determine the extent of illness using objective criteria, as compared to the PED clinicians who may not have performed testing. This may have also decreased the chance of parents returning to the ED if they felt that prior testing excluded certain pathologies or they better understood the exact reason for the illness. It is also possible that there was a difference in the anticipatory guidance provided to families or some other factor that may have impacted the return rates. Additionally, prior research has shown that children with complex or chronic conditions are more likely to revisit EDs, a factor that may disproportionately affect return rates at PEDs.^14^

The most notable differences in revisit rates between general EDs and PEDs were observed for conditions such as head/neck trauma, seizure, asthma, fever, and congestion/URI (Table 2). Of these, seizures and fevers could potentially be related to medical complexity, although these could not be determined definitively (as noted above). Only congestion/URI and fevers were in the top five initial presenting diagnoses. The reason for this variation is unclear but does persist across multiple diagnoses. It is unclear whether this was a reflection of care received in a general ED, the comfort of PED clinicians with discharging patients on initial visit, or some combination of factors.

Whether additional testing in a particular ED setting constitutes optimal initial care for both the patient and the medical system depends on the balance between the consequences of overtesting (such as cost, time, and radiation exposure) and the risks of missed pathology, including the outcomes of an ED revisit. Additionally, it is notable that there was a higher percentage of children < 6 years of age seen in PEDs when compared to general EDs in this study, which may have contributed to pediatric emergency clinicians being more comfortable sending younger patients home on the initial visit given the comparative volume of young children that are seen in general EDs. Balancing the value of this initial discharge with higher return rates also merits further investigation (Table 1).

For the most acute cases, care outcomes appeared similar, with comparable death rates for ESI 1 patients in general EDs and PEDs (5.1% vs 4.9%). However, the significantly higher discharge rate of ESI 1 patients in general EDs (60.9% vs 14% in PEDs) raises questions about discrepancies in initial triage based on the ED staff’s comfort level in dealing with children or something intrinsic to the patient population that presents to the different EDs. Recent research suggests that 15% of lower acuity cases (ESI 4 and 5) were undertriaged in general EDs while over half of ESI 3 and 4 level visits were overtriaged in the general ED and that mistriage was more common in general EDs than in PEDs.^16^ This points to the possibility that initial triage in the respective settings may ultimately impact 72-hour return visits. Future research should further explore these factors and their implications for pediatric emergency care quality.

## LIMITATIONS

Our primary challenge was the limitation of the dataset itself, because we had no way to identify patients who may not have re-presented to the same ED. It is possible that patients may have returned for their second visit to a PED rather than a general ED depending on local hospital availability and patient preferences, which may have skewed the results. That being said, we may safely assume that patients with high-acuity presentations would likely have returned to the closest ED, even if it was the same general ED they had previously visited.

In addition, this dataset encompasses only one large national emergency medicine physician group, and it is subject to data quality and reporting issues intrinsic to that group and its system. The hospitals included in this study represent a diverse network of institutions and patients across a wide variety of geographic regions. The proportion of children treated in pediatric EDs is much smaller when compared to general EDs in our dataset, but this reflects the national landscape that most PED visits do not occur in the pediatric setting, but rather in a community ED.

## CONCLUSION

Contrary to our initial hypothesis, we found that pediatric patients discharged from pediatric EDs had a higher rate of 72-hour return visits than those discharged from general EDs. Further research is needed to identify whether this difference is due to systems factors, patient factors, or differences in the quality of care to identify best practices and optimize care across ED settings.

## Figures and Tables

**Table 1:** Characteristics of all index visits.

	General EDs		Pediatric EDs		Difference (%)
	
	No.	%	No.	%	
Index visit characteristics					
Sex					
Female	892,076	49	366,563	47.8	−1.2
Male	929,724	51	400,317	52.2	1.2
Ages, years					
<1	180,854	9.9	118,871	15.5	5.6
1–3	430,888	23.7	227,387	29.7	6
4–6	285,734	15.7	132,930	17.3	1.6
7–9	230,692	12.7	96,393	12.6	−0.1
10–12	222,370	12.2	78,585	10.2	−2
13–15	251,864	13.8	69,277	9	−4.8
16–17	219,398	12	43,437	5.7	−6.4
Payor source					
Commercial	425,540	23.4	128,138	16.7	−6.6
Medicaid	1,156,229	63.5	570,169	74.3	10.9
Medicare	2,610	0.1	447	0.1	−0.1
Self-pay	191,737	10.5	58,506	7.6	−2.9
Other payor	45,615	2.5	9,549	1.2	−1.3
ESI Level					
3	482,697	26.5	239,536	31.2	4.7
4	921,245	50.6	392,488	51.2	0.6
5	95,905	5.3	102,114	13.3	8.1
Missing	321,953	17.7	32,742	4.3	−13.4
Clinician type					
Physician	1,039,835	57.1	589,143	76.8	19.7
APP	763,688	41.9	176,054	23	−19
Size of facility					
<30,000	309,708	17	183,191	23.9	6.9
30,000–59,999	1,057,728	58.1	95,353	12.4	−45.6
60,000+	454,364	24.9	488,336	63.7	38.7
Location of facility					
Large central metro	451,828	24.8	219,007	28.6	3.8
Large fringe metro	538,795	29.6	68,494	8.9	−20.6
Medium metro	454,734	25	95,353	12.4	−12.5
Small metro and non-metro	376,443	20.7	384,026	50.1	29.4
Primary Diagnoses					
Congestion/Upper respiratory infection	344,052	18.9	170,177	22.2	3.3
Trauma unspecified	235,890	12.9	71,570	9.3	−3.6
Extremity pain or injury	189,916	10.4	51,984	6.8	−3.6
Other diagnosis	119,939	6.6	55,385	7.2	0.6
Fever	114,089	6.3	71,859	9.4	3.1
Abdominal pain	117,554	6.5	65,235	8.5	2.1
Ear complaints	110,702	6.1	39,073	5.1	−1
Rash	101,931	5.6	40,961	5.3	−0.3
Head/Neck trauma	84,063	4.6	24,651	3.2	−1.4
Vomiting	63,400	3.5	36,981	4.8	1.3
Respiratory (other)	58,657	3.2	23,101	3	−0.2
Psychological/behavioral	16,739	0.9	2,631	0.3	−0.6
Urinary symptoms	32,448	1.8	11,042	1.4	−0.3
Asthma/wheezing	29,025	1.6	21,686	2.8	1.2
Eye complaints	29,673	1.6	11,772	1.5	−0.1
Headache	22,034	1.2	9,892	1.3	0.1
Chest pain	16,956	0.9	6,592	0.9	−0.1
Foreign body	16,086	0.9	6,233	0.8	−0.1
Poisoning	13,152	0.7	3,052	0.4	−0.3
Genitourinary - female	14,682	0.8	5,052	0.7	−0.1
Chronic disease	12,896	0.7	7,183	0.9	0.2
Allergic reaction	12,161	0.7	2,697	0.4	−0.3
Seizure	10,817	0.6	8,165	1.1	0.5
Fainting/syncope	9,849	0.5	3,477	0.5	−0.1
Genitourinary - male	8,441	0.5	4,198	0.5	0.1
Neurologic (other)	7,882	0.4	3,825	0.5	0.1
Pregnancy	7,453	0.4	481	0.1	−0.3
Cardiac complaints	5,533	0.3	1,972	0.3	0
Primary care	5,048	0.3	790	0.1	−0.2
Abuse/assault	4,547	0.2	1,056	0.1	−0.1
Gastrointestinal bleeding	2,403	0.1	1,608	0.2	0.1
Poor feeding	2,041	0.1	1,453	0.2	0.1
Respiratory	1,206	0.1	632	0.1	0
Device complication	519	0	394	0.1	0
General/unspecified symptoms	15	0	19	0	0
N	1,821,800	100	766,880	100	

*ED*, emergency department; *ESI*, Emergency Severity Index; *ESI*, Emergency Severity Index; *APP*, advanced practice privider.

**Table 2:** Percentage of index visits that returned to the same emergency department within 72 hours.

	General EDs		Pediatric EDs			[95% CI]
	
	Total Visits	% Returned	Total Visits	% Returned	Difference (%)	
Index visit characteristics						
Sex						
Female	892,076	2.4	366,563	3.5	1	[0.8 to 1.2]
Male	929,724	2.3	400,317	3.6	1.2	[0.9 to 1.5]
Ages, y						
<1	180,854	3.6	118,871	4.9	1.4	[0.8 to 1.9]
1–3	430,888	2.7	227,387	3.7	1	[0.7 to 1.4]
4–6	285,734	2.1	132,930	3.1	1	[0.6 to 1.4]
7–9	230,692	1.9	96,393	2.9	1	[0.7 to 1.4]
10–12	222,370	1.8	78,585	2.9	1	[0.7 to 1.3]
13–15	251,864	2	69,277	2.8	0.7	[0.5 to 1]
16–17	219,398	2.6	43,437	3.3	0.7	[0.4 to 1]
Payor source						
Commercial	425,540	1.9	128,138	3.1	1.2	[0.9 to 1.5]
Medicaid	1,156,229	2.6	570,169	3.7	1.1	[0.8 to 1.3]
Medicare	2,610	2.5	447	2.5	0	[−1.5 to 1.4]
Self-pay	191,737	2.1	58,506	2.9	0.9	[0.1 to 1.1]
Other payor	45,615	2.7	9,549	3.2	0.6	[0.5 to 1.2]
ESI level						
3	482,697	3.2	239,536	4.5	1.3	[0.9 to 1.8]
4	921,245	2.1	392,488	3.2	1.1	[0.7 to 1.5]
5	95,905	1.7	102,114	2.5	0.8	[0.6 to 1.1]
Missing	321,953	2.3	32,742	3.4	1.1	[0.8 to 1.3]
Clinician type						
Physician	1,039,835	2.6	589,143	3.6	1	[0.7 to 1.3]
APP	763,688	2	176,054	3.1	1	[0.8 to 1.2]
Size of facility						
<30,000	309,708	2.3	183,191	3.5	1.3	[0.6 to 1.9]
30,000–59,999	1,057,728	2.4	95,353	3.6	1.1	[0.8 to 1.4]
60,000+	454,364	2.4	488,336	3.5	1.1	[0.9 to 1.3]
Location of facility						
Large central metro	451,828	1.9	219,007	3.7	1.8	[1.4 to 2.2]
Large fringe metro	538,795	2.2	68,494	2.9	0.7	[0.5 to 0.9]
Medium metro	454,734	2.8	95,353	3.6	0.7	[0.2 to 1.2]
Small metro and non-metro	376,443	2.7	384,026	3.5	0.8	[0.6 to 1]
Primary Diagnoses						
Congestion/upper respiratory infection	344,052	2.9	170,177	3.8	1	[0.6 to 1.3]
Fever	114,089	3.7	71,859	4.8	1.1	[0.7 to 1.5]
Abdominal pain	117,554	3.3	65,235	4.2	0.9	[0.6 to 1.3]
Rash	101,931	3.3	40,961	4	0.8	[0.5 to 1.1]
Other dx	119,939	2.3	55,385	3	0.7	[0.5 to 1]
Trauma unspecified	235,890	1.1	71,570	2	0.8	[0.5 to 1.1]
Ear complaints	110,702	2.1	39,073	2.4	0.3	[0.1 to 0.4]
Extremity pain or injury	189,916	1.2	51,984	2.1	0.9	[−0.2 to 1.9]
Vomiting	63,400	3.3	36,981	4.2	0.9	[0.4 to 1.3]
Respiratory (other)	58,657	2.8	23,101	3.4	0.7	[0.1 to 1.2]
Head/neck trauma	84,063	1.3	24,651	3.5	2.1	[−1.3 to 5.6]
Urinary symptoms	32,448	3.3	11,042	4.5	1.2	[0.9 to 1.5]
Psychological/behavioral	16,739	2.8	2,631	3	0.3	[−0.7 to 1.3]
Asthma/wheezing	29,025	2.8	21,686	4.4	1.6	[1.1 to 2]
Headache	22,034	2.6	9,892	3.6	1	[0.6 to 1.4]
Seizure	10,817	3.9	8,165	6.1	2.2	[1.6 to 2.8]
Eye complaints	29,673	1.9	11,772	2.6	0.8	[0.4 to 1.1]
Chronic disease	12,896	3.2	7,183	3.9	0.8	[0.1 to 1.5]
Pregnancy	7,453	6	481	6	0	[−3.3 to 3.3]
Genitourinary - female	14,682	2.7	5,052	2.9	0.2	[−0.3 to 0.7]
Allergic reaction	12,161	2.7	2,697	3.9	1.1	[0.1 to 2.1]
Chest pain	16,956	1.6	6,592	2.2	0.6	[0.2 to 1.1]
Poisoning	13,152	1.3	3,052	2.3	1	[0.6 to 1.3]
Neurologic (other)	7,882	2.3	3,825	4.2	2	[1.1 to 2.8]
Foreign body	16,086	1.2	6,233	1.9	0.7	[0.2 to 1.2]
Genitourinary – male	8,441	1.8	4,198	2.3	0.4	[−0.1 to 0.9]
Cardiac complaints	5,533	2.7	1,972	3	0.3	[−0.5 to 1.1]
Fainting/syncope	9,849	1.6	3,477	2.1	0.5	[0.1 to 0.9]
Abuse/assault	4,547	1.6	1,056	1.7	0.1	[−0.7 to 1]
Poor feeding	2,041	3.1	1,453	4.1	1	[0.1 to 2]
Primary care	5,048	1.2	790	1.3	0.1	[−0.7 to 0.8]
Respiratory	1,206	4.1	632	3.5	−0.7	[−3 to 1.7]
Gastrointestinal bleeding	2,403	2.1	1,608	4	2	[1.1 to 2.9]
Device complication	519	3.9	394	5.8	2	[−0.6 to 4.6]
General/unspecified symptoms	15	6.7	19	5.3	−1.4	[−16.2 to 13.4]

*ED*, emergency department; *ESI*, Emergency Severity Index; *dx*, diagnosis; *APP*, advanced practice practitioner.
